# Impact of the oxidative balance score on cardiovascular-kidney-metabolic syndrome: A cross-sectional study with machine learning prediction

**DOI:** 10.1371/journal.pone.0334050

**Published:** 2025-10-09

**Authors:** Xin Liu, Li Rong, Haoting He

**Affiliations:** 1 Cardiovascular Department, The Affiliated Traditional Chinese Medicine Hospital, Guangzhou Medical University, Guangzhou, Guangdong, China; 2 Cardiovascular Department, The First Affiliated Hospital of Guangzhou University of Chinese Medicine, Guangzhou, Guangdong, China; The First Affiliated Hospital of Soochow University, CHINA

## Abstract

**Background and aim:**

The antioxidant diet and lifestyle are widely believed to prevent and even treat various diseases; however, their applicability to cardiovascular-kidney-metabolic (CKM) syndrome remains unknown. In this study, the correlation between the oxidative balance score (OBS) and CKM syndrome was investigated, along with the underlying mechanisms involved.

**Methods and results:**

The study data were obtained from participants with complete OBS and diagnostic information on CKM syndrome in the NHANES from 1999 to 2018. The correlation between OBS and the incidence of CKM syndrome was investigated by weighted multivariable logistic regression analysis. Mediation analyses were conducted to explore the mediating role of inflammatory markers in the relationship between these two variables. A restricted cubic spline graph and threshold effects were constructed to identify nonlinear associations. OBS was applied to eight machine learning algorithms to develop a predictive model and assess its performance. A total of 30113 participants were included in our study. OBS was negatively correlated with CKM syndrome regardless of covariate adjustment. Compared with the lowest quartile of OBS, the highest quartile resulted in a 33% decrease in the incidence of CKM syndrome. A nonlinear association was identified using a restricted cubic spline, with 30 being the threshold point. Mediation analysis indicated that high-sensitivity C-reactive protein (hsCRP) and systemic immune inflammation (SII) partially inﬂuenced the relationship between these two parameters. Among the eight machine learning algorithms, XGBoost presented the highest area under the curve (AUC), demonstrating superior predictive performance and clinical efficacy.

**Conclusions:**

The prevalence of CKM syndrome was negatively correlated with an increase in OBS, which may be partially related to its anti-inflammatory effect.

## 1. Introduction

The prevalence of obesity, diabetes, cardiovascular disease (CVD), and chronic kidney disease (CKD) is increasing in developed countries and is a serious threat to public health. According to the WORLD HEART REPORT 2023 released by the World Heart Federation (WHF), approximately 20.5 million individuals are projected to die from CVD in 2021 [1]. A Global Burden of Disease study revealed that the worldwide incidence of CKD was approximately 9.1% in 2017, accompanied by a significant increase in mortality of 41.5% across all age groups [2]. Furthermore, the global prevalence of diabetes among the adult population was estimated to be 10.5% through 2021, with projections indicating a 20% increase by 2045 [3]. The intricate network of interrelated health disorders has recently been defined as cardiovascular‒kidney‒metabolic (CKM) syndrome by the American Heart Association (AHA) [4]. A complicated interaction of hemodynamic and neurohormonal mechanisms, including sympathetic hyperactivity, activation of the renin‒angiotensin‒aldosterone system, oxidative stress, and persistent chronic inflammation, was characterized as the pathogenesis of CKM syndrome [5]. An imbalance between the generation of reactive oxygen species and the antioxidant defense system is classified as oxidative stress, which is intimately linked to CVD, CKD, and metabolic syndrome (MetS) [6–8]. Adjusting the diet structure and therapeutic lifestyle changes may effectively prevent CKM syndrome by reducing oxidative stress and improving the inflammatory internal environment.

The oxidative balance score (OBS) is a valid and reliable observational survey of the effects of oxidation on diet and lifestyle that summarizes the balance between five pro-oxidants and fifteen antioxidants [9]. Previous studies have shown that a high OBS was negatively related to the prevalence of ischemic heart disease [10], CKD [11], type 2 diabetes [12], and mortality [13]. Additionally, numerous studies have demonstrated relationships between three major chronic diseases caused by CKM syndrome and various antioxidants, including vitamin C, vitamin E, zinc, and folate [14–17]. To our knowledge, the relationship between OBS and CKM syndrome has not been explored. In our study, we evaluated the potential relationship between OBS and CKM syndrome to prevent the onset of CKM syndrome through improvements in dietary structure and lifestyle habits.

## 2. Methods

### 2.1. Data sources and study population

The National Center for Health Statistics (NCHS) performs the nationally representative National Health and Nutrition Examination Survey (NHANES) to evaluate the health and nutritional status of the general population in the United States. The data employed for this cross-sectional analysis were from this program. To increase the representativeness of the study, sample weights were utilized to rectify disparities in selection probabilities, compensate for potential deficiencies within eligible samples, and account for noncoverage and nonresponse.

**Fig 1:**
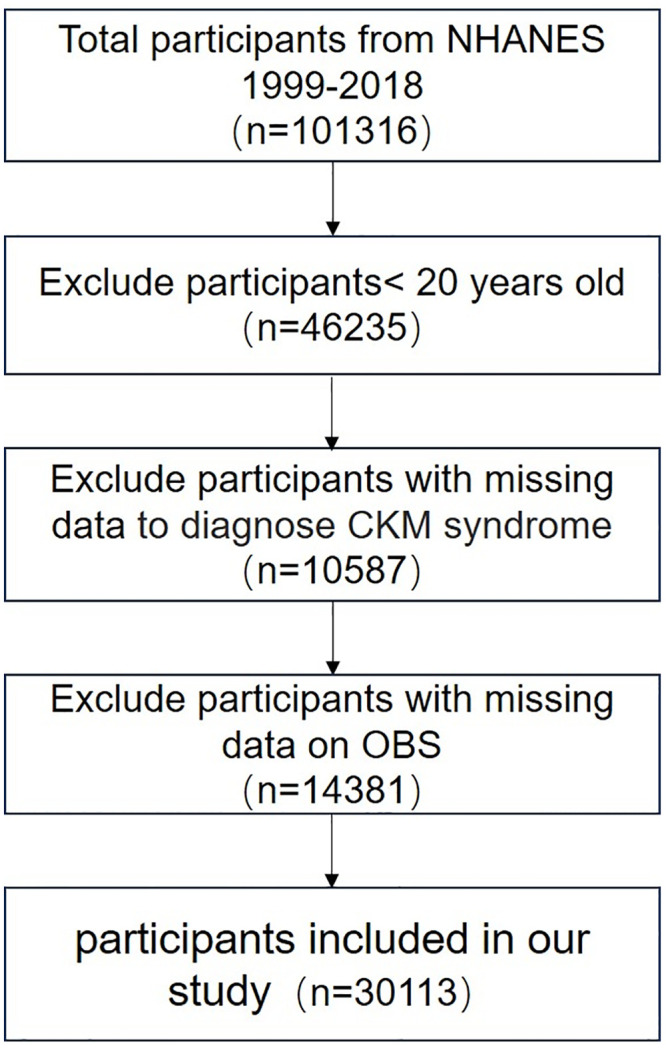
The flowchart of study participants’ selection.

The data from the NHANES comprised a total of 101,317 valid participants and were collected and merged over ten cycles from 1999 to 2018; however, the authors could not access information that could identify individual participants during or after data collection. The following exclusion criteria were applied in this study: (1) participants <20 years old, (2) participants with missing data on OBS scoring rules, and (3) participants lacking data to diagnose CKM syndrome. As a result, 30113 eligible participants were included in the final analysis of our study, whose detailed ﬂowchart is shown in Fig 1.

### 2.2. Exposure and outcomes

The main exposure in our study was OBS, namely, a modified version developed by Goodman [18]. The questionnaire consisted of twenty components, sixteen of which were dietary factors and four of which were lifestyle factors; the detailed criteria are shown in S1 Table. The dietary food intake data for each participant in the NHANES dataset were documented through a two-day 24-hour dietary recall interview. The initial dietary recall was conducted in person at the Mobile Examination Center, followed by a second interview via telephone 3 to 10 days later. Daily average intakes of nutrient components were based on a two-day dietary recall interview. Lifestyle data, including physical activity, body mass index (BMI), alcohol consumption, and smoke exposure, were collected and calculated by weekly metabolic equivalent (MET) scores, body measurement data, the average daily quantity of alcoholic beverages consumed over the past 12 months via an alcohol use questionnaire, and serum cotinine levels, respectively. A decrease in the scores of the five pro-oxidants and an increase in the scores of the fifteen antioxidants scores induced elevated OBS. The detailed results regarding these observations are presented in S1 Table.

CKM syndrome staging was initially introduced by Chiadi E.Numele at the AHA in November 2023, and was defined as the construct aimed at reflecting pathophysiological progression within the CKM syndrome spectrum and increasing the absolute risk of CVD [19]. Stage 4 was the most severe period of CKM syndrome, associated with higher mortality rates and clinical interventions. The outcome of our study was stage 4 CKM syndrome, which was classified as clinical CVD (including coronary heart disease(CHD), heart failure, heart attack, angina, and stroke), accompanied by excess adipose tissue, dysfunctional adipose tissue (including obesity, abdominal obesity, impaired fasting glucose and impaired glucose tolerance), CKM syndrome risk factors (hypertriglyceridemia (≥135 mg/dL)), hypertension, metabolic syndrome, or type 2 diabetes), or CKD.

### 2.3. Covariates

To mitigate potential confounding biases, numerous covariates were identified based on clinical research. These included age, ethnic group, gender, educational background, marital status, and the poverty-to-income ratio (PIR) as categorical variables (<1, 1–3, or > 3), daily energy intake and risk factors for CKM syndrome, including hypertension, hyperlipidemia, MetS, CKD and type 2 diabetes, were also indentified. The detailed classification criteria used are available at https://www.cdc.gov/nchs/nhanes (accessed April 10, 2024).

### 2.4. Statistical analysis

In this study, Mobile Examination Center (MEC) exam weights were employed for data analysis. Continuous variables including age, PIR, daily energy intake, and OBS were calculated by weighted linear regression models and are summarized as the means with standard deviations (SDs), whereas categorical variables including other demographic covariates and history of related diseases were examined through weighted chi-square tests and are presented as proportions. To analyze the correlation between OBS and the incidence of CKM syndrome, weighted multivariate linear regression analysis was conducted, and the OBS was additionally divided into quartiles as a categorical variable to further investigate the relationship. The variance inflation factor (VIF) was used to measure the multicollinearity among the covariates. To mitigate multicollinearity, covariates with a VIF exceeding 5 were removed from the analysis. In Model 1, no adjusted covariates were performed; Model 2 was adjusted for demographic confounders, including gender, race, educational level, marital status, age, and PIR; and Model 3 was adjusted for all covariates. To investigate the mediating function of inflammatory indicators, mediation analysis was performed. To further examine the association between OBS and CKM syndrome, a restricted cubic spline graph (RCS) was selected for this study because of its interpretability and flexibility in capturing nonlinear relationships without a predefined parametric form [20]. In addition, a threshold effect analysis was conducted to identify potential inflection points in the association. To confirm the stability of our study, sensitivity analysis and stratiﬁed analyses were also performed.

Incomplete covariate data above 15% were excluded from our study, whereas missing values of covariates below 15% were addressed using multiple imputations. In the subgroup analysis, which was stratified on the basis of each covariate, the model did not include adjustments for the variable itself. Statistical analysis was performed with R-version 4.1.1. By transforming the categorical variables into continuous variables, the P for trend was calculated. A p-value less than 0.05 was considered to indicate statistical significance.

### 2.5. Prediction model establishment and validation

To increase the efficiency of the machine learning and clinical prediction models, variables were identified using the least absolute shrinkage and selection operator (LASSO), excluding covariates with coefficients equal to 0. The prediction model included demographic factors, comorbidities, and OBS variables while excluding other blood indicators obtained invasively. The dataset was randomly divided into a training set and a testing set, with 70% allocated for training and the remaining 30% assigned to testing set. To ensure the robustness of the model, the training set was subjected to fivefold cross-validation, iterative testing, and fine-tuning to develop the most effective model. Models were designed with a training set aimed at predicting the risk of CKM syndrome, while their efficacy was evaluated with a testing set. In this study, eight algorithms, namely, logistic regression (LR), random forest (RF), elastic net (ENET), extreme gradient boosting (XGBoost), support vector machine (SVM), decision tree (DT), K-nearest neighbors (KNN), and multilayer perceptron (MLP), were employed to analyze the selected variables.

The performance of all the models was evaluated by the area under the curve (AUC) of the receiver operating characteristic (ROC) curve, accuracy, recall, and precision. Calibration curves were used to evaluate the accuracy of the models in predicting absolute risk, and decision curve analysis (DCA) demonstrated the models’ net benefits at various thresholds, reflecting their practical value in clinical decision-making. To improve the clinical utility and predictive accuracy of the models, an online risk calculator with superior performance was developed.

## 3. Results

### 3.1. Baseline characteristics

The proportion of missing data for each variable was illustrated in S1 Fig, which indicates that multiple interpolations were applied to missing values for all confounders. Additionally, S2 Table presents the variance inflation factors for each variable, suggesting that multicollinearity was absent among the variables. The weighted baseline characteristics are shown in Table 1. Patients were divided into two groups according to the diagnosis of CKM syndrome: 2556 had CKM syndrome, and 27,557 did not. In total, all participants in our study who were diagnosed with CKM syndrome had an average age of 47.80 ± 17.44 years. The distribution of sex was as follows: 50.59% male and 49.41% female. The ethnic proportions were as follows: 16.9% Mexican-American, 19.06% non-Hispanic Black, 48.78% non-Hispanic White, 7.56% other Hispanic, and 8.52% others. Compared with participants without CKM syndrome, those with CKM syndrome had significantly different demographic factors. The individuals who were diagnosed with CKM syndrome were more likely to be elderly, male, and non-Hispanic White, and to have a lower income, lower education level, lower daily energy intake, and lower OBS scores.

**Table 1:** Characteristics of the study population based on the prevalence of CKM syndrome.

Variable	Overall (n = 30113)	No CKM syndrome (n = 27557)	CKM syndrome (n = 2556)	P-value
Age, years	47.80 ± 17.44	44.86 ± 15.68	63.51 ± 13.04	<0.0001
PIR				<0.0001
<1	17.73	17.57	19.44	
1-3	39.97	39.45	45.58	
>3	42.43	42.98	34.98	
Energy, kcal/day	2048.28 ± 956.61	2105.13 ± 947.73	1936.41 ± 862.59	<0.0001
OBS	20.69 ± 7.07	21.61 ± 6.94	19.60 ± 6.93	<0.0001
Gender				<0.0001
Male	50.59	49.53	60.09	
Female	49.41	50.47	39.91	
Race				<0.0001
Mexican American	16.09	7.45	3.90	
Non-Hispanic Black	19.06	9.62	9.25	
Non-Hispanic White	48.78	71.55	78.88	
Other Hispanic	7.56	5.06	2.66	
Other Race – Including Multi-Racial	8.52	6.31	5.31	
Marital status				<0.0001
Married/Living with a partner	19.10	15.45	27.89	
Widowed/divorced/separated	62.81	66.02	66.21	
Never married	18.09	18.53	5.89	
Education				<0.0001
Less than high school	8.48	3.68	6.62	
High school	35.36	31.26	41.37	
More than high school	56.16	65.06	52.02	
Diabetes				<0.0001
No	78.63	83.96	57.08	
Yes	14.33	9.36	31.92	
IFG	4.36	4.09	7.54	
IGT	2.69	2.58	3.46	
METS				<0.0001
No	69.98	73.86	41.45	
Yes	30.02	26.14	58.55	
CKD				<0.0001
No	85.12	89.68	66.11	
Yes	14.88	10.32	33.89	
Hypertension				<0.0001
No	61.54	68.60	25.48	
Yes	38.46	31.40	74.52	
Hyperlipidemia				<0.0001
No	28.79	31.46	9.99	
Yes	71.21	68.54	90.01	

Mean ± SD for PIR, age, energy, and OBS.

N% for: gender, race, marital status, educational background, hypertension, metabolic syndrome (MetS), CKD, diabetes, hyperlipidemia.

### 3.2. Correlation between OBS and CKM syndrome

The correlation between OBS and CKM syndrome in our study is illustrated in Table 2 by multivariate regression models, and p-values were retained to four decimal places and rounded. According to the crude model (Model 1), the odds ratio (OR) 95% confidence interval (CI), and P-value were 0.97 (0.96–0.98; P < 0.001), indicating a negative association between continuous OBS and the incidence of CKM syndrome. The relationships remained consistent between Model 2 (OR (95% CI): 0.95(0.95,0.97), P < 0.001) and the fully adjusted Model (Model 3) (OR (95% CI): 0.97(0.96,0.98), P < 0.001). When OBS was categorized by quartile, we observed a negative correlation between the highest quartile of OBS and CKM syndrome (OR (95% CI): 0.67 (0.55, 0.82)) in Model 3, compared with the lowest level of OBS (Q1). Moreover, signiﬁcant associations were also found in the 2^nd^ and 3^rd^ quartile OBSs in Models 2 and 3. The trend test between these quartiles remained significant across all three models (p for trend <0.05). The relationships among lifestyle, dietary OBS, and CKM syndrome are also shown in Table 2. Dietary and lifestyle OBSs were shown to be significantly connected with a lower risk of CKM syndrome when considered as continuous factors in the fully adjusted Model 3. When the data were converted to categorical variables, higher dietary or lifestyle OBSs were associated with a lower incidence of CKM syndrome in all the models, except for the 2^nd^ quartile of lifestyle OBS.

**Table 2:** Association of oxidative balance score and CKM syndrome.

	Model 1	Model 2	Model 3
	OR (95%CI) P-value	OR (95%CI) P-value	OR (95%CI) P-value
OBS	0.96(0.95, 0.97) <0.0001	0.97 (0.96, 0.98) <0.0001	0.97 (0.96, 0.98) <0.0001
Q1	Ref.	Ref.	Ref.
Q2	0.81(0.70, 0.94) 0.0050	0.82 (0.70, 0.96) 0.0169	0.83 (0.70, 0.99) 0.0360
Q3	0.61(0.51, 0.72) <0.0001	0.68 (0.56, 0.81) 0.001	0.71 (0.58, 0.87) 0.0014
Q4	0.50(0.43, 0.58) <0.0001	0.58 (0.50, 0.69) <0.0001	0.67 (0.55, 0.82) 0.0002
P for trend	<0.0001	<0.0001	0.001
Dietary OBS	0.96(0.95,0.97) <0.0001	0.97 (0.97, 0.98) <0.0001	0.98 (0.97, 0.99) 0.0003
Q1	Ref.	Ref.	Ref.
Q2	0.79 (0.68, 0.91) 0.0015	0.82 (0.70, 0.97) 0.0198	0.82 (0.69, 0.98) 0.0333
Q3	0.57 (0.49, 0.68)<0.0001	0.66 (0.56, 0.79) <0.0001	0.69 (0.57, 0.84) 0.0003
Q4	0.51 (0.45, 0.59)<0.0001	0.65 (0.56, 0.76) <0.0001	0.71 (0.58, 0.86) 0.0009
P for trend	<0.0001	<0.0001	0.004
Lifestyle OBS	0.88 (0.85, 0.91)<0.0001	0.80 (0.77, 0.83) <0.0001	0.87 (0.84, 0.91) <0.0001
Q1	Ref.	Ref.	Ref.
Q2	0.95 (0.80, 1.12) 0.5506	0.86 (0.71, 1.04) 0.1236	0.89 (0.72, 1.08) 0.2392
Q3	0.84 (0.72, 0.99) 0.0352	0.65 (0.54, 0.77) <0.0001	0.75 (0.63, 0.90) 0.0027
Q4	0.65 (0.55, 0.76)<0.0001	0.46 (0.39, 0.54) <0.0001	0.61 (0.51, 0.73) <0.0001
P for trend	<0.0001	<0.0001	<0.0001

Model 1 was adjusted for none.

Model 2 was adjusted for demographic covariates

Model 3 was adjusted for all covariates

### 3.3. Restricted cubic spline graph and threshold effect analysis

**Fig 2:**
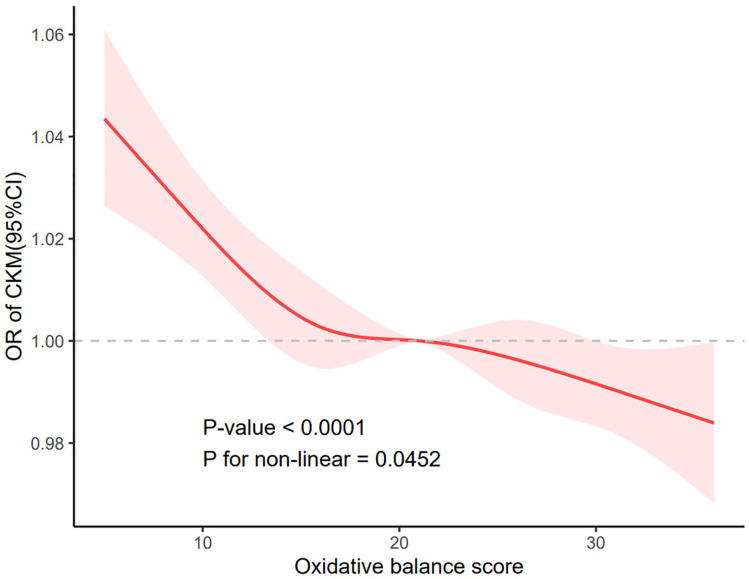
RCS results for the association between OBS and the prevalence of CKM syndrome.

A restricted cubic spline was used to verify whether the negative association between OBS and the incidence of CKM syndrome was linear (Fig 2). A significant nonlinear association was observed between the two variables after adjusting for all confounders (P for nonlinearity < 0.05). Further analysis was conducted to explore the inflection point in the relationship between OBS and CKM syndrome (Table 3). In the fully regulated fitted graphs, inflection points were identified, reaching a critical value of 30. Moreover, for each unit increase in OBS, there was a significant 12% reduction in the prevalence of CKM syndrome. Below the threshold of 30, the protective effect appeared to stabilize.

**Table 3:** Threshold effect analysis of oxidative balance score on CKM syndrome.

	OBS	Dietary OBS	Lifestyle OBS
Model 1			
Linear effect model	0.98 (0.97, 0.98) <0.001	0.98 (0.97, 0.99) <0.001	0.90 (0.87, 0.93) <0.001
Model 2			
Inflection point(K)	30	10	2
< K	0.98 (0.97, 0.99) <0.001	0.95 (0.92, 0.98) 0.002	0.98 (0.80, 1.19) 0.81
> K	0.88 (0.80, 0.97) 0.01	0.99 (0.98, 1.00) 0.02	0.89 (0.86, 0.93) <0.001
Log-likelihood ratio	0.029	0.052	0.408

### 3.4. Interaction analysis and stratiﬁed analyses

**Fig 3:**
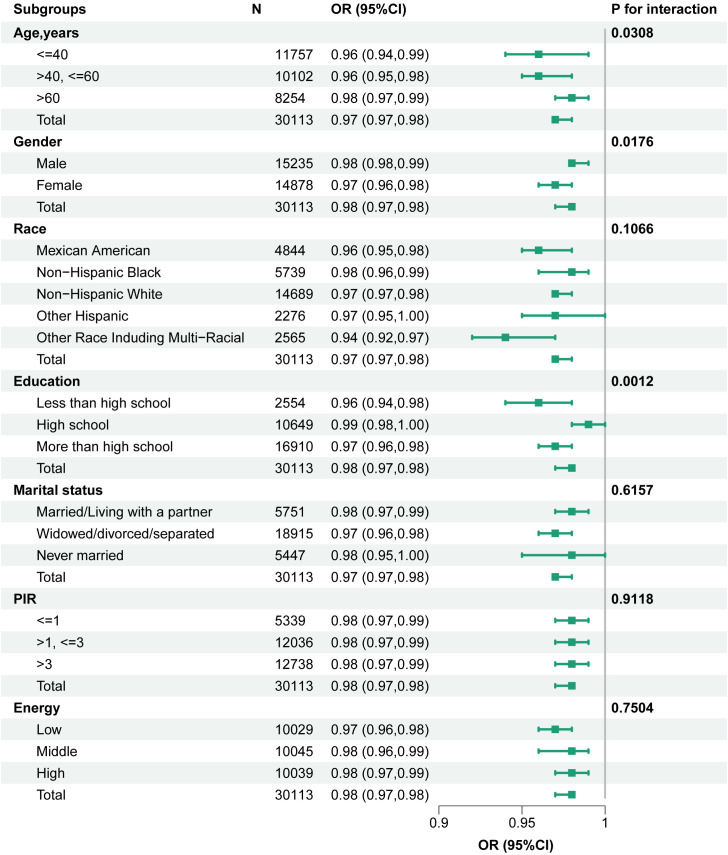
Stratiﬁed analyses for association between OBS and prevalence of CKM syndrome. Models were adjusted for all covariates, excluding the stratiﬁcation factor itself.

The stratified analyses and forest plots, which visualize the OR (95% CI) as depicted in Fig 3, consistently illustrated that higher OBS values were associated with a decreased prevalence rate of CKM syndrome across the majority of subgroups. No significant association was detected between OBS and CKM syndrome within the subgroup of individuals with an ethnicity of other Hispanic and individuals with a high school education. Interaction term analysis revealed that the association was significantly influenced by educational background (P for interaction = 0.001), gender (P for interaction = 0.017), and age when categorized at thresholds of 40 and 60 years (P for interaction = 0.031).

### 3.5. Mediation analyses

Inflammation plays a crucial role in CKM syndrome. A mediation analysis was conducted to evaluate whether the correlation between OBS and the incidence of CKM syndrome could be mediated by inflammatory biomarkers, including high-sensitivity C-reactive protein (hsCRP), leucocyte (WBC), systemic immune-inflammation (SII), and the lymphocyte‒monocyte ratio (LMR). The results presented in Table 4 indicate that hsCRP and the SII significantly positively mediate this association, accounting for 5.7% and 2.7% of the effect, respectively. This suggested that hsCRP and the SII partially inﬂuenced the relationship between the two.

**Table 4:** Mediation effects of inflammatory factors on the association between OBS and CKM syndrome.

Mediator	Total effect		Indirect effect		Direct effect		Mediated proportion,%
	β(95%CI)	P value	β(95%CI)	P value	β(95%CI)	P value	
hsCRP	−0.025589 (−0.040830-,-0.009615)	<0.001	−0.001466 (−0.002739, −0.000260	0.0140	−0.024(−0.039644,-0.008269)	0.0040	5.7%
WBC	−0.016989 (−0.022091, −0.011268)	<0.001	−0.000312 (−0.000677,0)	0.0500	−0.016677 (0.021778, −0.010868)	<0.001	1.8%
SII	−0.017095 (−0.022658, −0.011268)	<0.001	−0.000468(−0.000756, −0.000170)	<0.001	−0.016627(−0.022340,-0.010752)	<0.001	2.7%
LMR	−0.018180(−0.035903,-0.001421)	<0.001	0 (−0.000146, 0.000252)	1	−0.018180(−0.036026, −0.001372)	<0.001	0

### 3.6. Sensitivity analyses

Owing to the application of multiple interpolation methods to address the missing values of confounders in this study, a sensitivity analysis was performed to verify the stability of our findings. A total of 26,621 eligible participants were included in the sensitivity analyses after excluding all individuals with incomplete data. As indicated in S3 Table, OBS remained negatively associated with the risk of CKM syndrome, which demonstrated the robustness of our study.

### 3.7. Establishment and validation of the prediction model

**Fig 4:**
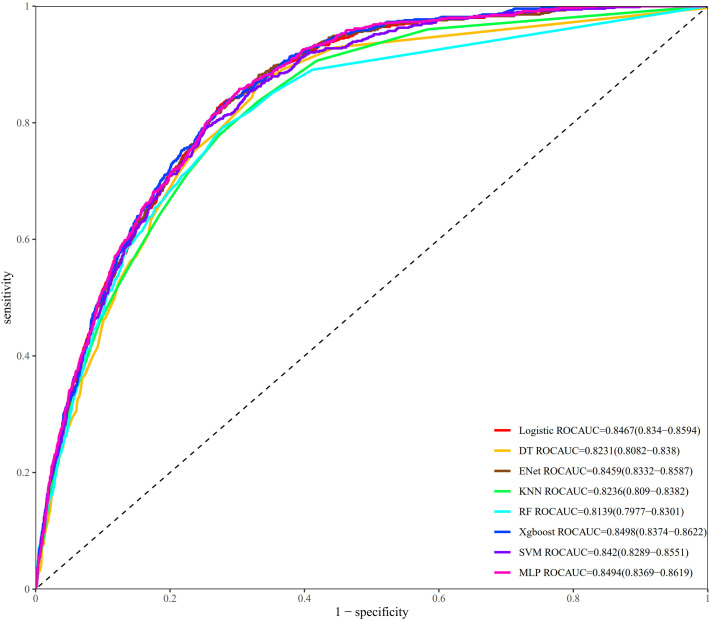
ROC curves on testing data of eight machine learning algorithm models.

**Fig 5:**
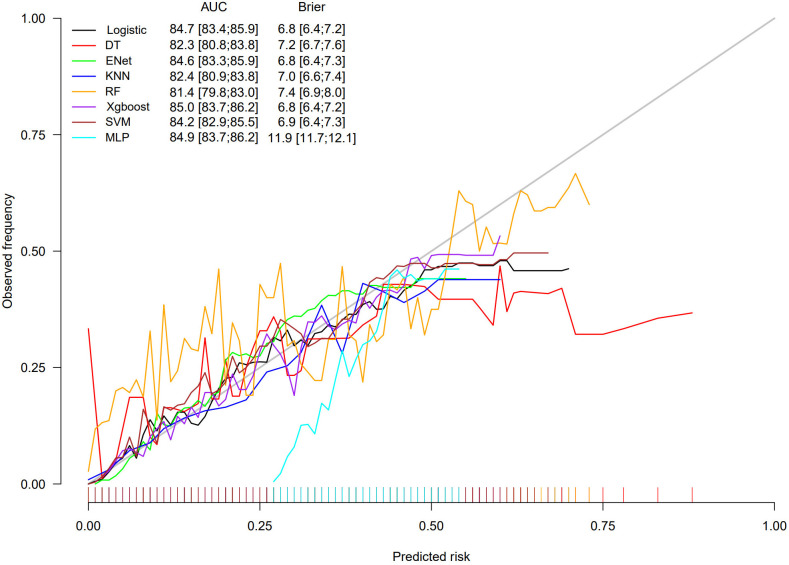
Calibration curves on testing data of eight machine learning algorithm models.

**Fig 6:**
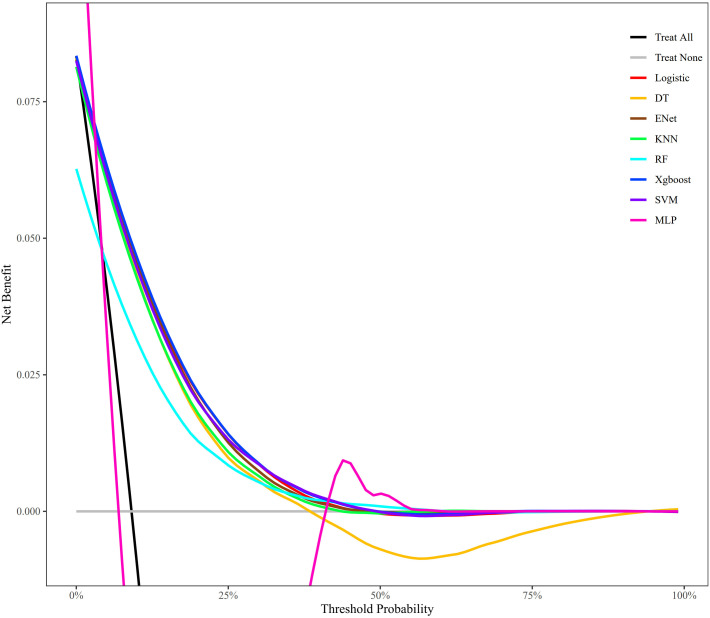
DCA curves on testing data of eight machine learning algorithm models.

The results of the LASSO model including the coefficient path and cross-validation curve are presented in S2 and S3 Figs. Following the principle of straightforward exploration, variables including OBS, age, gender, hypertension, CKD, MetS, and diabetes were incorporated into eight machine-learning algorithm models. To demonstrate the consistency and robustness of the cross-validation results, the mean AUC and standard deviation from the five-fold cross-validation of each model are shown in S4 Fig. The ROC curves of the models are shown in Fig 4, in which the AUC values of all the models exceeded 0.8. XGBoost and MLP presented the highest AUC values, at 0.850 and 0.849, respectively. The AUC values for the other models were as follows: LR (0.847), RF (0.814), DT (0.823), SVM (0.842), KNN (0.824), and Enet (0.846). The accuracies of all the models were as follows: LR (0.770), RF (0.754), DT (0.761), SVM (0.765), KNN (0.752), Enet (0.770), XGBoost (0.774), and MLP (0.775). The precision values for all the models were as follows: LR (0.209), RF (0.220), DT (0.202), SVM (0.218), KNN (0.197), Enet (0.210), XGBoost (0.227) and MLP (0.221). The recall values for all the models were as follows: LR (0.854), RF (0.773), DT (0.844), SVM (0.813), KNN (0.837), Enet (0.851), XGBoost (0.817) and MLP (0.834). To avoid model overfitting, hyperparameter tuning had already been performed. However, owing to the large sample size and the relatively low proportion of positive cases of CKM syndrome in the dataset, class imbalance led to low precision. Among all the models tested, XGBoost achieved the highest AUC value and highest precision, indicating superior discriminatory ability and better accuracy in identifying positive cases. After balancing precision and recall, these results suggest that XGBoost is the most effective model for predicting CKM syndrome, offering robust and generalizable performance across multiple dimensions. Fig 5 shows the calibration curves of the LR, Enet, SVN, and XGBoost algorithms closely approximated the ideal line, indicating high accuracy of the predicted probabilities. According to the DCA, XGBoost showed the greatest net benefits across multiple threshold ranges, suggesting significant advantages in clinical applications (Fig 6). However, owing to its robust performance, high efficiency, and ability to automatically handle complex relationships among features of XGBoost, particularly structured clinical data, the XGBoost model was used in this study to develop an online risk calculator.

Using the Shiny platform, we created a web application based on the model to make clinical prediction models easier for researchers and clinicians to utilize (https://phoenixlx.shinyapps.io/xgboost_ckm/). The risk of CKM syndrome can be predicted by entering the relevant information into the appropriate web interface location.

## 4. Discussion

In our study, a significant nonlinear negative relationship was observed between OBS and the risk of developing CKM syndrome, which remained consistent even after adjustments for all potential confounders. Threshold effect analysis identified 30 as the inflection point, above this point, the protective effect of OBS appeared to be more significant. Moreover, interaction analysis demonstrated more significant differences in the subgroups defined by gender, age, and educational background. The hsCRP and the SII, which are markers of inflammation, serve as partial mediators of this relationship. Furthermore, eight machine learning algorithms for variables, including OBS, were used to construct clinical prediction models. The XGBoost model demonstrated the highest predictive accuracy among all tested models, with an AUC of 0.850, the highest precision (0.227), and high accuracy (0.774). These results highlight its superior ability to correctly classify the incidence of CKM syndrome and support its robustness as a predictive tool. However, this web application is only applicable for predicting the occurrence of stage 4 CKM syndrome. As a multi-stage disease, CKM syndrome may exhibit different relationships with OBS at other stages. Future studies should expand the analysis to earlier stages and offer valuable insights into the potential role of OBS in the early detection and management of CKM syndrome.

In the initial stage of CKM syndrome, excess and dysfunctional adiposity along with insulin resistance play central roles in the pathogenesis of CKM syndrome. Compared with regular adipose tissue, abdominal adipose tissue has a greater level of phagocytes which leads to the emergence of oxidative stress, severe inflammatory infiltration, and excessive free radical production when hypoxic-ischemic necrotic adipocytes are phagocytosed [21–23]. Interconnected molecular mechanisms, such as hyperglycemia, insulin resistance, elevated renin‒angiotensin‒aldosterone system (RAAS) activity, oxidative stress, lipotoxicity, impaired mitochondrial function, and persistent chronic inflammation, are regarded as the pathophysiology of CKM syndrome. It is crucial to focus on prevention, management, and therapy to improve health outcomes in patients with CKM syndrome [24]. The majority of risk factors associated with CKM syndrome, such as overweight, smoking, excessive alcohol use, poor dietary habits, and physical inactivity, are related to lifestyle and are modifiable. Obesity is acknowledged to be a significant factor that contributes to the increased prevalence of CKM syndrome and cardiovascular events [25]. Therefore, making extensive therapeutic lifestyle adjustments, namely, adjustments that prioritize enhancing physical activity, regulating diet, and promoting weight reduction, has been identified as a crucial strategy for controlling and reducing the incidence of CKM syndrome.

Lifestyle modification has been shown to have a beneficial effect on risk factors that contribute to the progression of CVD, obesity, diabetes mellitus, and hyperlipidemia [26,27]. OBS can be used to quantitatively assess the impact of diet and lifestyle habits on health from an antioxidative perspective. Total fat, serum iron, alcohol consumption, BMI, and serum cotinine are categorized as pro-oxidants, whereas dietary fiber, serum carotene, riboflavin, niacin, total folate, vitamin B6, vitamin B12, vitamin C, vitamin E, calcium, magnesium, zinc, copper, selenium and exercise metabolism equivalents (METs) are classified as antioxidants; this classification implies that higher levels of antioxidants and lower levels of pro-oxidants correspond to elevated OBS. A healthy dietary structure, characterized by reduced intake of high-calorie foods and moderate intake of plant-based foods, is recommended because of its role in mitigating dyslipidemia and dysglycemia, as well as preventing ectopic fat distribution to peripheral organs [28]. Dietary adjustments are correlated with a decreased prevalence of tissue lipotoxicity, oxidative stress, and inflammation, all of which can negatively impact the health of important organs, including the kidneys, liver, and heart. The intake of vegetables and fruits, which are rich in nutrients, particularly vitamin C, folate, and dietary fiber, as well as in phosphorus, potassium, and magnesium, is negatively correlated with CVD, hypertension, type 2 diabetes mellitus, obesity, CKD and other cardiometabolic risk factors for CVD, which are major diagnostic criteria for CKM syndrome [29–31]. Moreover, incorporating vitamin E into a high-fat fructose diet was found to reduce cholesterol levels, lower blood pressure, and potentially meliorate disorders accompanying MetS in hereditary hypertriglyceridemic rats [32]. However, a review and meta-analysis suggested that while high intake of folic acid, vitamin B6, and vitamin B12 offered cardiovascular protective effects in the general population, these benefits did not extend to patients with clinical CVD or renal dysfunction [33]. Selenium has been shown to inhibit the synthesis of insulin-like growth factors to stimulate the insulin effect, decompose lipid peroxides, and regulate blood cholesterol levels and platelet aggregation to improve blood pressure through glutathione peroxidase (GSH-Px), which affects the prevalence and development of insulin resistance, diabetes and MetS [34,35]. A Korean NHANES-based cross-sectional study indicated that a dietary calcium intake exceeding 800 mg/day decreased the risk of CVD events in women who had been postmenopausal for more than ten years [36]. Obesity was identified as a primary factor in the progression of CKM syndrome, which highlights the importance of obesity management in reducing the risk of CKM syndrome. The first-line approach to treating obesity has transitioned from merely reducing BMI to a comprehensive lifestyle intervention strategy. This approach aims to decrease body fat, maintain or even increase muscle mass, and manage complications associated with obesity [37]. Intensive lifestyle interventions (ILIs) have been established as the primary strategy for the effective treatment of obesity and the management of associated cardiometabolic risk factors [38]. In summary, a higher OBS, which is calculated by the dietary and lifestyle composite score of sixteen pro-/antioxidant components mentioned above, is inversely related to the risk factors for CKM syndrome and significantly reduces the 10-year atherosclerotic cardiovascular disease (ASCVD) risk [39]. Furthermore, our study indicates that elevated OBS is associated with a lower prevalence of CKM syndrome and explores the underlying mechanisms, which may be related to its anti-inflammatory effects.

Inflammation and oxidative stress play crucial roles in the development of CVD, CKD, and MetS, which are essential diagnostic elements of CKM syndrome. The hsCRP is a plasma protein produced in response to inflammatory stimuli such as hyperglycemia and hyperlipidemia, and is recognized as a classical inflammatory biomarker. Elevated levels of hsCRP are closely associated with atherosclerosis and coronary heart disease, which highlights the necessity of anti-inflammatory treatment in the prevention of cardiovascular events [40]. The SII, a novel and stable inflammatory marker, effectively reflects both local immune and systemic inflammatory responses. First defined in 2014, the SII is calculated as the platelet count ×neutrophil count/lymphocyte count [41]. Previous studies have indicated that the predictive value of the SII for major cardiovascular events following coronary intervention is significantly superior to that of traditional risk factors [42]. Our study revealed that OBS was inversely correlated with the SII and hsCRP, suggesting that elevated OBS levels may be linked to reduced inflammatory states in the body. Similarly, Hinako Nanri reported that elevated OBSs were inversely associated with oxidative stress and inflammation indicators in Japanese adults [43]. A NHANES-based study also explored the correlation between OBS and MASLD mediated by systemic inflammation, with the results demonstrating that elevated OBS levels may be related to lower SII and IR values [44]. The potential reason is that OBS scoring criteria include a wide range of micronutrients and in which zinc, magnesium, carotenoids, and nicotinamide play crucial roles in maintaining internal homeostasis, reducing C-reactive protein (CRP) and indicators of oxidative stress, promoting pancreatic β-cell antioxidant capacity and decreasing the prevalence of T2DM, METs, and CKD [45–48].

Although hsCRP and the SII show statistically significant partial mediation effects between OBS and CKM syndrome, their effect proportions are only 5.7% and 2.7%, respectively. This finding suggests that inflammation may only partially explain the link between OBS and CKM syndrome, indicating the involvement of additional or more proximal mediating mechanisms. Oxidative stress is closely associated with disturbances in insulin signaling pathways, mitochondrial dysfunction and dyslipidemia, which are central features of CKM syndrome but are not necessarily mediated by systemic inflammation. A relatively high OBS may reduce cardiovascular death in individuals with diabetes and prediabetes by improving insulin resistance [20]. Another study suggested that engaging in regular physical exercise, including aerobic training, resistance training, or combined training can promote antioxidative stress, improve lipid metabolism, enhance mitochondrial function, decrease MetS scores, and preserve renal function [49–51]. Since CKM syndrome is a newly defined disease, the underlying mechanism that mediates the interaction between OBS and CKM syndrome remains to be further explored.

Interestingly, the negative relationship between OBS and CKM syndrome was of greater magnitude in females, highly educated individuals, and younger participants, indicating that the effect of OBS on CKM syndrome might be significantly influenced by demographic characteristics. Previous research has indicated that males are more likely to be smokers, averse to plant-based diets, have central obesity, and under great mental pressure, which are crucial risk factors contributing to the sex difference in mortality [52]. However, females presented a greater prevalence of CKD and a lower incidence of CVD than males did at all stages of CKD. These differences were less significant among patients undergoing dialysis or transplantation [53]. The sample size in some subgroups was small, which may have resulted in insufficient statistical data to detect a weak association. Although there were no statistically significant differences for individuals with a high school education level or individuals with an ethnicity of other Hispanic, there may be other uncontrolled confounding factors (e.g., lifestyle factors, genetic background, mental health) that could affect the statistical relationship between OBS on CKM syndrome; for example, numerous studies have indicated that compared with college graduates, individuals with lower educational levels have higher weight, incidence of CVD outcomes among type 2 diabetes (T2D) patients, and ten-year risk of ASCVD [54,55]. Age is a high-risk factor for various chronic diseases, especially cardiovascular events [56]. Although the prevalence of CKM syndrome may be influenced by multiple confounding factors, our findings have been supported by numerous clinical and basic studies showing that the risk of CKM syndrome may be significantly lower in people who maintain an antioxidant-rich diet and antioxidant-promoting lifestyle, especially women, young adults, and highly educated individuals.

### 4.1. Effect on clinical practice

Guided by the findings of recent research, clinicians continue to rely on tools developed for CVD and renal disorders, most notably the Framingham Risk Score, the ASCVD risk assessment, and the American Heart Association’s updated PREVENT equations, the latter of which incorporate additional variables—including the UACR and hemoglobin concentration—to refine cardiovascular risk stratification in patients with CKD [57,58]. As a newly recognized clinical syndrome, CKM syndrome currently lacks a dedicated risk-prediction algorithm. In our study, the results of exploring the connection between OBS and CKM syndrome revealed that OBS might be a useful predictive indicator. Using an internet risk calculator can help investigators efficiently assess the risk of developing CKM syndrome, which can lead to timely lifestyle adjustments and treatment of high-risk factors to improve patient prognosis. Compared with conventional risk‐scoring algorithms, the XGBoost model achieved the AUC values of 0.850, indicating great discriminative power for both case classification and risk prediction [59]. Subsequent studies should subject the model to cross-validation and independent external cohorts to rigorously assess its calibration and temporal stability and identify which specific components of the OBS most accurately predict the development of CKM syndrome.

### 4.2. Research limitations

Our study has several limitations. First, subjective factors may introduce measurement errors and influence the accuracy of dietary intake assessments. Second, owing to the small sample size of data for some variables in the adolescent population, only adults older than 20 years were selected for this study, and the results do not apply to minors. Third, despite adjustments for various variables in the statistical analyses, there may be potential confounders that were not accounted for in our study. Fourth, this study focuses on stage 4 CKM syndrome, which may limit the generalizability of our findings.

## 5. Conclusion

In conclusion, our findings indicate that elevated levels of OBS within a specific range are linked to a decreased incidence of CKM syndrome. To further study this potential mechanism, additional RCTs or cross-sectional studies are necessary to verify this outcome to provide more promising, effective, and simple prevention and treatment methods to improve the prognosis of CKM syndrome patients.

## Supporting information

S1 Table Oxidative balance scoring criteria. (DOCX)

S2 Table Variance inflation factors for all variables. (DOCX)

S3 Table Sensitivity analysis between OBS and CKM syndrome. (DOCX)

S4 Table List of abbreviations. (DOCX)

S1 Fig Missing value percentage for variables. (TIF)

S2 Fig Coefficients path of LASSO model. (TIF)

S3 Fig Cross validation curve of LASSO model. (TIF)

S4 Fig The mean AUC and standard deviation from the five-fold cross-validation of eight models. (TIF)
