# Supplementary material for: Impact of the oxidative balance score on cardiovascular-kidney-metabolic syndrome: A cross-sectional study with machine learning prediction
Source: PLoS One. 2025 Oct 9;20(10):e0334050. doi: 10.1371/journal.pone.0334050 (PMC12510519; doi:10.1371/journal.pone.0334050)
Supplement: S1 Table — (DOCX) [file pone.0334050.s001.docx]

| OBS components | Property | Male | | | Female | | |
| --- | --- | --- | --- | --- | --- | --- | --- |
|  |  | 0 | 1 | 2 | 0 | 1 | 2 |
| Dietary OBS components | | | | | | | |
| Dietary fiber(g/d) | A | <12.56 | 12.56-19.70 | ≥19.70 | <10.10 | 10.10-16.31 | >16.31 |
| Carotene(RE/d) | A | <98.83 | 98.83-306.25 | ≥306.25 | <98.08 | 98.08-383.50 | ≥383.5 |
| Riboflavin(mg/d) | A | <1.79 | 1.79-2.69 | ≥2.69 | <1.34 | 1.34-2.02 | ≥2.02 |
| Niacin(mg/d) | A | <20.65 | 20.65-29.75 | ≥29.75 | <14.52 | 14.52-21.86 | ≥21.86 |
| Vitamin B6(mg/d) | A | <1.59 | 1.59-2.40 | ≥2.40 | <1.13 | 1.13-1.77 | ≥1.77 |
| Total folate(mcg/d) | A | <316.00 | 316.00-492.00 | ≥492.00 | <251.00 | 251.00-388.96 | ≥388.96 |
| Vitamin B12(mcg/d) | A | <3.36 | 3.36-6.20 | ≥6.20 | <2.22 | 2.22-4.22 | ≥4.22 |
| Vitamin C(mg/d) | A | <42.44 | 42.44-113.21 | ≥113.21 | <38.01 | 38.01-98.49 | ≥98.49 |
| Vitamin E(mg/d) | A | <5.82 | 5.82-9.42 | ≥9.42 | <4.53 | 4.53-7.52 | ≥7.52 |
| Calcium(mg/d) | A | <646.00 | 646.00-1072.00 | ≥1072.00 | <499.24 | 499.24-849.00 | ≥849.00 |
| Magnesium(mg/d) | A | <257.00 | 257.00-361.28 | ≥361.28 | <187.00 | 187.00-283.43 | ≥283.43 |
| Zinc(mg/d) | A | <9.75 | 9.75-15.10 | ≥15.10 | <6.73 | 6.73-10.75 | ≥10.75 |
| Copper(mg/d) | A | <1.12 | 1.12-1.57 | ≥1.57 | <0.85 | 0.85-1.28 | ≥1.28 |
| Selenium(mg/d) | A | <94.94 | 94.94-141.80 | ≥141.80 | <67.79 | 67.79-99.50 | ≥99.50 |
| Total fat(g/d) | P | ≥69.83 | 69.83-107.43 | <107.43 | ≥50.98 | 50.98-75.79 | <75.79 |
| Iron(mg/d) | P | ≥12.88 | 12.88-19.17 | <19.17 | ≥9.65 | 9.65-14.32 | <14.32 |
| Lifestyle OBS components | | | | | | | |
| Physical activity(MET-minute/week) | A | <417.86 | 417.86-1135.71 | ≥1135.71 | <270.00 | 270.00-845.71 | ≥845.71 |
| Alcohol(g/d) | P | ≥30 | 0-30 | None | ≥15 | 0-15 | None |
| BMI(kg/m^2^) | P | ≥25.54 | 25.54-29.17 | <29.17 | ≥23.74 | 23.74-28.64 | <28.64 |
| Cotinine(ng/ml) | P | ≥0.038 | 0.038-1.13 | <1.13 | ≥0.035 | 0.035-0.172 | <0.172 |

Table S1 Oxidative Balance Scoring Criteria

A:antioxidant, P:prooxidant, RE: retinol equivalent; ATE: alpha-to-copherol equivalent, MET: metabolic equivalent.
