# Supplementary material for: Impact of the oxidative balance score on cardiovascular-kidney-metabolic syndrome: A cross-sectional study with machine learning prediction
Source: PLoS One. 2025 Oct 9;20(10):e0334050. doi: 10.1371/journal.pone.0334050 (PMC12510519; doi:10.1371/journal.pone.0334050)
Supplement: S2 Table — (DOCX) [file pone.0334050.s002.docx]

| Variable | VIF |
| --- | --- |
| Marital status | 1.3 |
| PIR | 1.2 |
| Education level | 1.3 |
| Hypertension | 1.4 |
| METS | 1.3 |
| CKD | 1.1 |
| Diabetes | 1.1 |
| Age | 1.7 |
| Gender | 1.2 |
| Race | 1.1 |
| Hyperlipidemia | 1.2 |
| Energy | 1.6 |
| OBS | 1.5 |

## Table S2 Variance inflation factors for all variables
