# Supplementary material for: Impact of the oxidative balance score on cardiovascular-kidney-metabolic syndrome: A cross-sectional study with machine learning prediction
Source: PLoS One. 2025 Oct 9;20(10):e0334050. doi: 10.1371/journal.pone.0334050 (PMC12510519; doi:10.1371/journal.pone.0334050)
Supplement: S3 Table — (DOCX) [file pone.0334050.s003.docx]

Table S3 Sensitivity analysis between OBS quartiles and CKM syndrome in all models

|  | Quartiles of OBS | | | | | OBS |
| --- | --- | --- | --- | --- | --- | --- |
|  | Q1（<15） | Q2  (15-21) | Q3  (21-26) | Q4  (>26) | P for trend |  |
| Model 1 | 1 | 0.81 (0.70, 0.93) | 0.63 (0.53, 0.76) | 0.51(0.44, 0.59) | <0.0001 | 0.96 (0.95, 0.97) |
| Model 2 | 1 | 0.83 (0.71,0.98) | 0.71 (0.58,0.86) | 0.60 (0.51,0.71) | <0.0001 | 0.97 (0.96,0.98) |
| Model 3 | 1 | 0.84 (0.70, 1.00) | 0.73 (0.59, 0.91) | 0.67 (0.55, 0.83) | 0.0002 | 0.97 (0.96, 0.98) |

Model 1 was adjusted for none

Model 2 was adjusted for demographic covariates

Model 3 was adjusted for all covariates
