# Supplementary material for: Impact of the oxidative balance score on cardiovascular-kidney-metabolic syndrome: A cross-sectional study with machine learning prediction
Source: PLoS One. 2025 Oct 9;20(10):e0334050. doi: 10.1371/journal.pone.0334050 (PMC12510519; doi:10.1371/journal.pone.0334050)
Supplement: S4 Table — (DOCX) [file pone.0334050.s004.docx]

Table S4 List of abbreviations

| **Full name** | **Abbreviations** |
| --- | --- |
| cardiovascular-kidney-metabolic | CKM |
| oxidative balance score | OBS |
| high-sensitivity c-reactive protein | hsCRP |
| systemic immune inflammation | SII |
| cardiovascular disease | CVD |
| chronic kidney disease | CKD |
| World Heart Federation | WHF |
| American Heart Association | AHA |
| metabolic syndrom | MetS |
| National Center for Health Statistics | NCHS |
| National Health and Nutrition Examination Survey | NHANES |
| acute myocardial impaction | AMI |
| body mass index | BMI |
| metabolic equivalent | MET |
| coronary heart disease | CHD |
| poverty-to-income ratio | PIR |
| standard deviation | SD |
| variance inflation factor | VIF |
| least absolute shrinkage and selection operator | LASSO |
| logistic regression | LR |
| random forest | RF |
| elastic net | ENET |
| extreme gradient boosting | XGBoost |
| support vector machine | SVM |
| decision tree | DT |
| K-nearest neighbors | KNN |
| multilayer perceptron | MLP |
| area under curve | AUC |
| receiver operating characteristic | ROC |
| odds ratio | OR |
| 95% confidence interval | 95% CI |
| lymphocyte-monocyte ratio | LMR |
| glutathione peroxidase | GSH-Px |
| intensive lifestyle interventions | ILIs |
| atherosclerotic cardiovascular disease | ASCVD |
