# Supplementary figures and images for: Impact of the oxidative balance score on cardiovascular-kidney-metabolic syndrome: A cross-sectional study with machine learning prediction
Source: PLoS One. 2025 Oct 9;20(10):e0334050. doi: 10.1371/journal.pone.0334050 (PMC12510519; doi:10.1371/journal.pone.0334050)

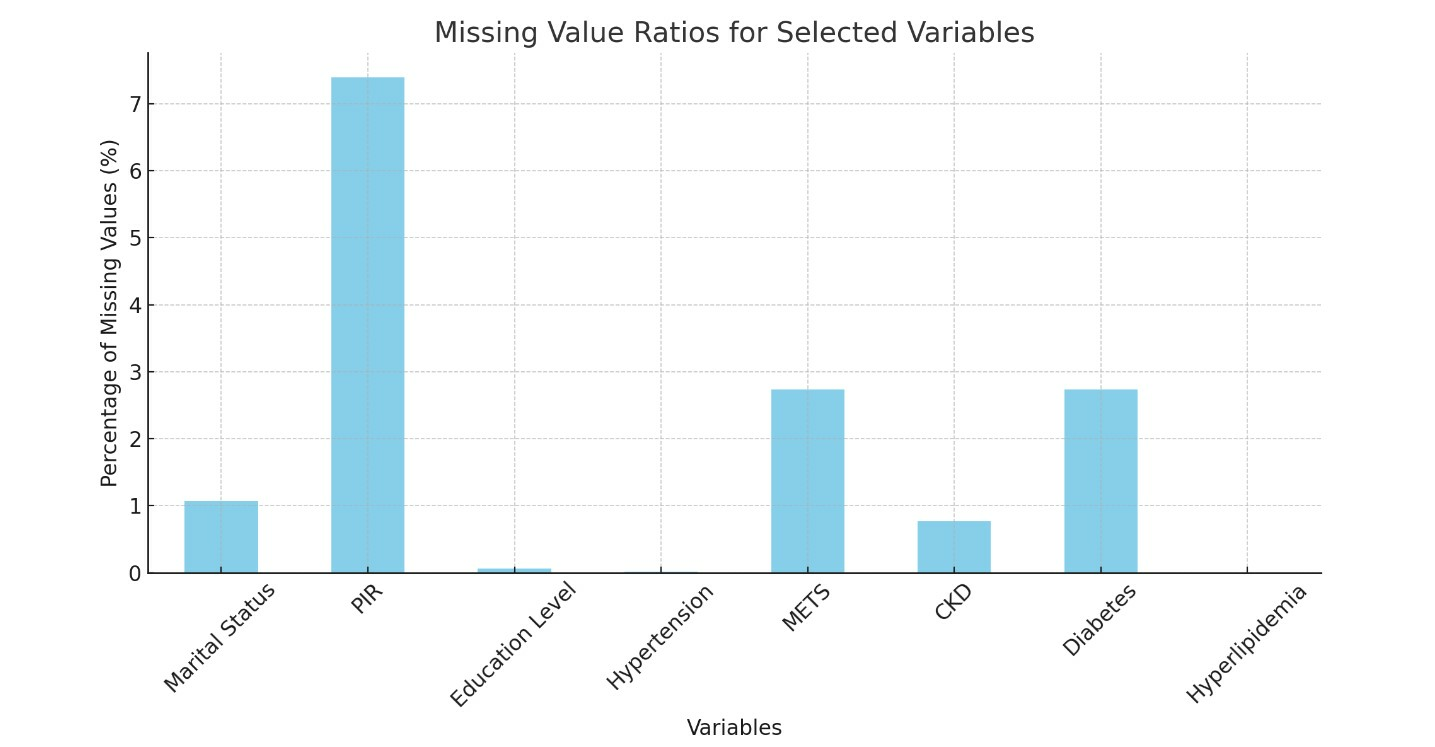

Supplement: S1 Fig — (TIF) [file pone.0334050.s005.tif]

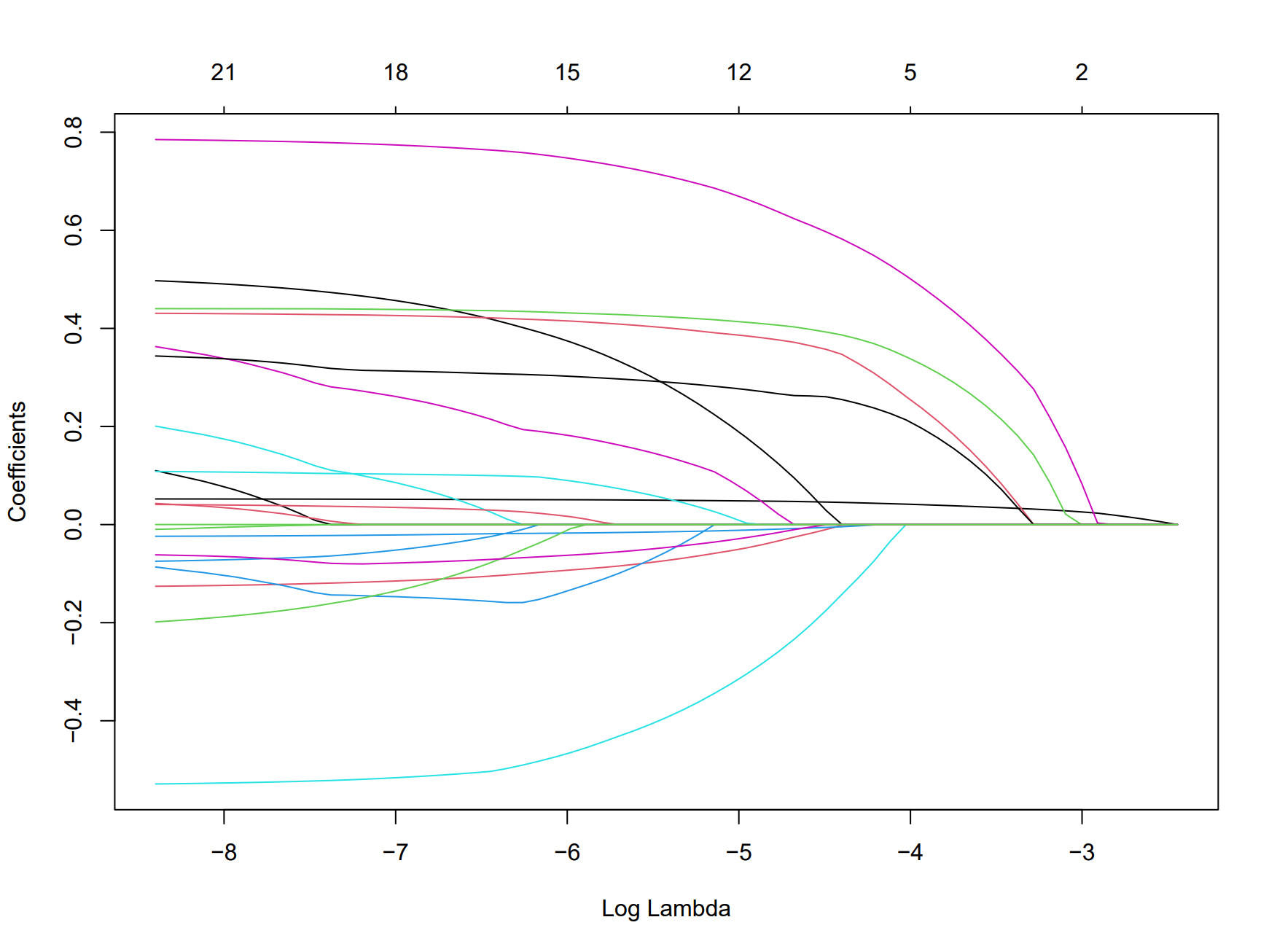

Supplement: S2 Fig — (TIF) [file pone.0334050.s006.tif]

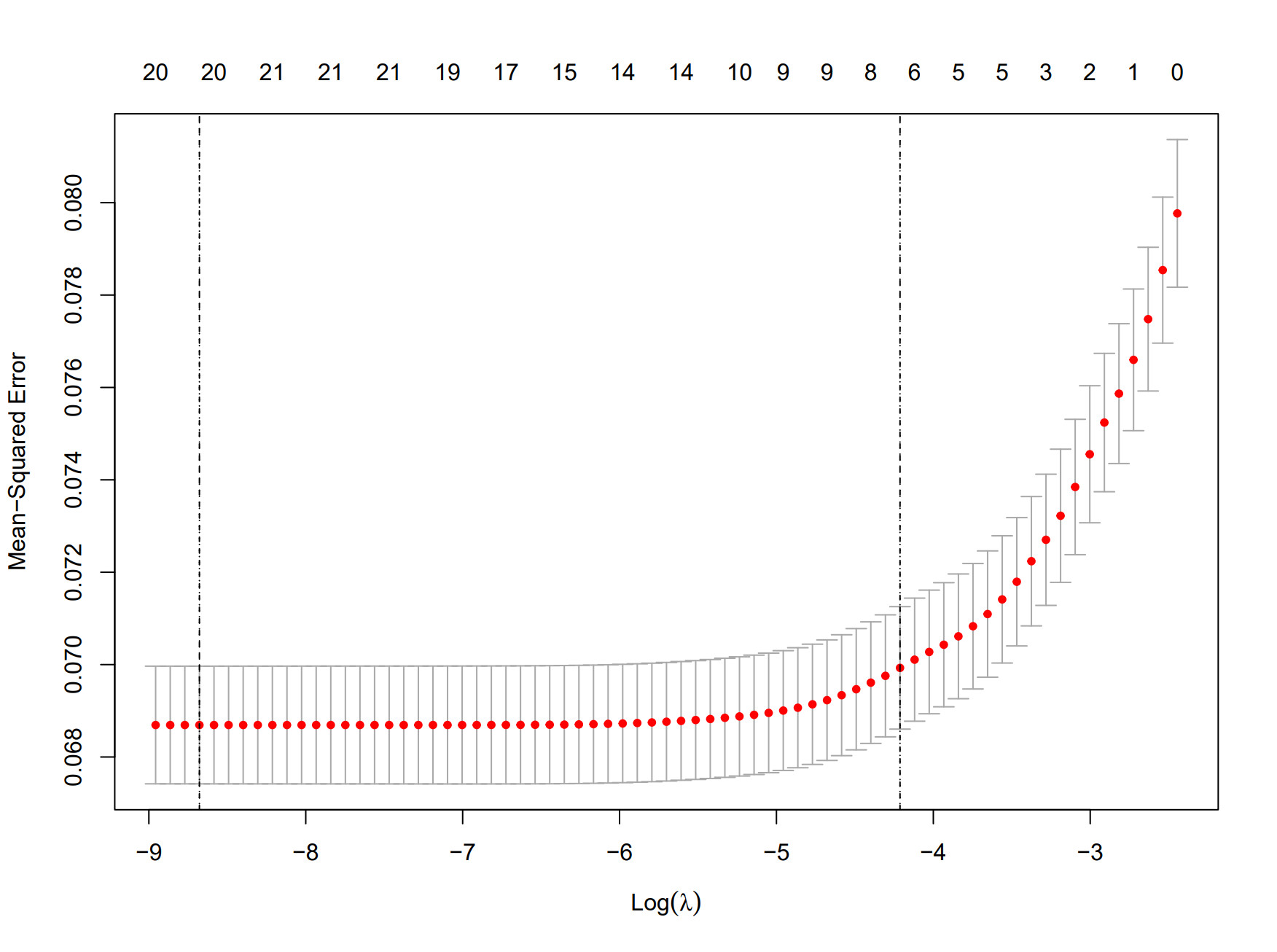

Supplement: S3 Fig — (TIF) [file pone.0334050.s007.tif]

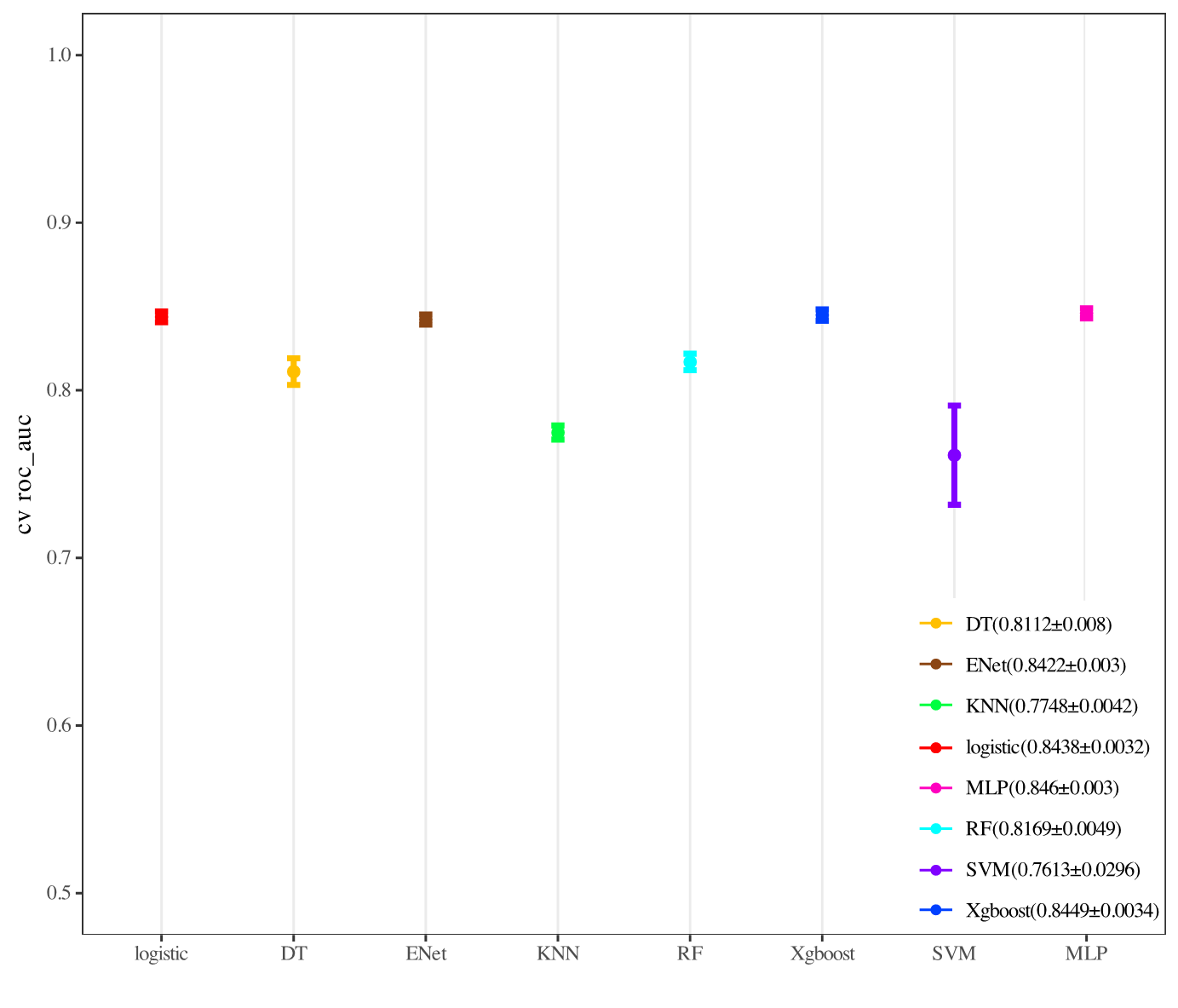

Supplement: S4 Fig — (TIF) [file pone.0334050.s008.tif]
